# Comprehensive Analysis of *BRCA1*, *BRCA2* and *TP53* Germline Mutation and Tumor Characterization: A Portrait of Early-Onset Breast Cancer in Brazil

**DOI:** 10.1371/journal.pone.0057581

**Published:** 2013-03-01

**Authors:** Dirce Maria Carraro, Maria Aparecida Azevedo Koike Folgueira, Bianca Cristina Garcia Lisboa, Eloisa Helena Ribeiro Olivieri, Ana Cristina Vitorino Krepischi, Alex Fiorini de Carvalho, Louise Danielle de Carvalho Mota, Renato David Puga, Maria do Socorro Maciel, Rodrigo Augusto Depieri Michelli, Eduardo Carneiro de Lyra, Stana Helena Giorgi Grosso, Fernando Augusto Soares, Maria Isabel Alves de Souza Waddington Achatz, Helena Brentani, Carlos Alberto Moreira-Filho, Maria Mitzi Brentani

**Affiliations:** 1 Laboratory of Genomics and Molecular Biology, A.C. Camargo Hospital, São Paulo, Brazil; 2 Radiology and Oncology Department, Faculdade de Medicina da Universidade de São Paulo, São Paulo, Brazil; 3 Laboratory of Structural Genomics, A.C. Camargo Hospital, São Paulo, Brazil; 4 Laboratory of Bioinformatics and Bioestatistics, A.C. Camargo Hospital, São Paulo, Brazil; 5 Department of Mastology, A.C. Camargo Hospital, São Paulo, Brazil; 6 Department of Oncogenetics, Hospital do Câncer de Barretos, São Paulo, Brazil; 7 Department of Mastology, Instituto Brasileiro de Controle do Câncer (IBCC), São Paulo, Brazil; 8 Department of Investigative Pathology, A.C. Camargo Hospital, São Paulo, Brazil; 9 Department of Molecular Oncogenetics, A.C. Camargo Hospital, São Paulo, Brazil; 10 Department of Psychiatry, Faculdade de Medicina da Universidade de São Paulo, São Paulo, Brazil; 11 Department of Pediatrics, Faculdade de Medicina da Universidade de São Paulo, São Paulo, Brazil; 12 National Institute of Science and Technology in Oncogenomics (INCITO), São Paulo, SP, Brazil; IFOM, Fondazione Istituto FIRC di Oncologia Molecolare, Italy

## Abstract

Germline mutations in *BRCA1*, *BRCA2* and *TP53* genes have been identified as one of the most important disease-causing issues in young breast cancer patients worldwide. The specific defective biological processes that trigger germline mutation-associated and -negative tumors remain unclear. To delineate an initial portrait of Brazilian early-onset breast cancer, we performed an investigation combining both germline and tumor analysis. Germline screening of the *BRCA1, BRCA2*, *CHEK2* (c.1100delC*)* and *TP53* genes was performed in 54 unrelated patients <35 y; their tumors were investigated with respect to transcriptional and genomic profiles as well as hormonal receptors and HER2 expression/amplification. Germline mutations were detected in 12 out of 54 patients (22%) [7 in *BRCA1* (13%), 4 in *BRCA2* (7%) and one in *TP53* (2%) gene]. A cancer familial history was present in 31.4% of the unrelated patients, from them 43.7% were carriers for germline mutation (37.5% in *BRCA1* and in 6.2% in the *BRCA2* genes). Fifty percent of the unrelated patients with hormone receptor-negative tumors carried *BRCA1* mutations, percentage increasing to 83% in cases with familial history of cancer. Over-representation of DNA damage-, cellular and cell cycle-related processes was detected in the up-regulated genes of *BRCA1/2*-associated tumors, whereas cell and embryo development-related processes were over-represented in the up-regulated genes of *BRCA1/2*-negative tumors, suggesting distinct mechanisms driving the tumorigenesis. An initial portrait of the early-onset breast cancer patients in Brazil was generated pointing out that hormone receptor-negative tumors and positive familial history are two major risk factors for detection of a *BRCA1* germline mutation. Additionally, the data revealed molecular factors that potentially trigger the tumor development in young patients.

## Introduction

Breast cancer in patients under the age of 35 y occurs in 2–10% of cases in Western countries, although this frequency may differ among different ethnic groups [1]–[5].

In Brazil, the incidence of breast cancer is high, with a trend of increased incidence among younger women since the 1980s. In the age range of 25–29 y, the rate increased from 6.4 to 7.8 per 100,000 women, while in the range of 30–34 y, the rate of incidence increased from 19 to 27.6 per 100,000 women [6]. This boost in early-onset breast cancer may be explained by either an increase in case notification or as a result of changes in the exposure pattern to different environmental risk factors [6]. Early-onset breast cancer is associated with worse outcome, despite aggressive therapies [1], [4], [5], [7]–[9]. Accordingly, invasive breast carcinomas in young patients exhibit clinical-biological characteristics of aggressive disease [8]–[11] and are associated with poor relapse-free survival [12]. This phenomenon can be partially attributed to the greater frequency of hormonal receptor/HER2-negative tumors in this group compared with late-onset breast cancer patients [12] in addition to poor differentiation, lymphovascular invasion and high proliferative fraction [10], [13].

Breast cancer has increasingly been described as a heterogeneous disease that displays a variety of subtypes with distinct gene expression profiles that have substantial implications for prognoses and survival rates [14]. It has been suggested that biological differences in tumors of early- and late-onset breast cancer patients are mainly influenced by expression profiles inherent to breast cancer subtype and grade [15].

The risk factors for early-onset breast cancer patients are still poorly understood; however, a familial history of cancer is a very important feature present in 10–37% of all cases. Among early-onset familial cases, 10–40% was found to be associated with *BRCA1* and *BRCA2* (*BRCA1/2*) mutations. In contrast, among sporadic early-onset breast cancer patients, the frequency of *BRCA1*/*2* mutation ranges from 1–10% [16]–[18]. Other susceptibility genes for breast cancer, such as *TP53, ATM*, *PALB2*, and the deletion at position 1100 of the *CHEK2* gene account for a small proportion of familial breast cancer patients [19]. Compelling data have shown that breast tumors from patients carrying germline *BRCA1*/*2* mutations are also morphologically and genetically different from each other as well as both sporadic and hereditary *BRCAx*-associated tumors. The last category is a heterogeneous group supposedly driven by mutation in as-yet unidentified genes [20]–[24].

The specific defective biological processes that trigger *BRCA1/2*-associated and -negative tumors remain unclear; whether tumorigenesis in early- and late-onset breast cancer patients differs is also unknown. Therefore, our main goals in the current study were to determine the mutation rate of the major breast cancer susceptibility genes in young Brazilian breast cancer patients and to characterize the immunohistochemical and molecular features of their tumors. We screened the *BRCA1, BRCA2*, *CHEK2 (c.del1100C)* and *TP53* genes for germline mutations in a cohort of 54 young women under the age of 35 y who developed breast cancer. We investigated their respective tumors with respect to hormonal receptors and HER2 status and compared the results with a cohort of 224 tumors of late-onset breast patients. We also assessed the transcriptional profiles of the tumors of the early-onset breast cancer patients. Additionally, we investigated the pattern of germline copy number variations (CNVs) and somatic acquired chromosomal alterations (SCNA) in a subset of matched samples. Taken together, the results permitted the outlining of a portrait of early-onset breast cancer in Brazil.

## Materials and Methods

### Patients

Patients were ascertained at three reference cancer centers in the state of São Paulo, Brazil: Hospital A. C. Camargo, São Paulo; Instituto Brasileiro de Controle do Câncer, IBCC, São Paulo; and Hospital do Câncer de Barretos, Barretos. All patients provided a written informed consent agreeing in participating in this study. All patients received genetic counseling. This study was approved by the Institutional Ethics Committee under number 818/06 (AC Camargo Hospital).

Fifty-four unrelated young patients with breast cancer diagnosed at an early age (35 y) were included in the study for germline mutation screening. The patients were classified on family history based on NCCN (www.nccn.org) criteria for Breast and Ovarian Cancer Syndrome. Tumor and blood samples were collected during biopsy or breast surgery. Peripheral blood of an affected sister diagnosed with breast cancer an age of 29 was used for confirming the germline alteration identified in the index patient. Patients received no neoadjuvant treatment before tumor and blood collection, with the exception of patient ID_2019. Two samples of peripheral blood (5 ml) were collected; fresh frozen tumor samples were submitted to histological analysis and manual dissection was performed by a pathologist. Only samples containing at least 70% malignant cells were included in the study. An additional group of tumor samples was derived from a cohort of 224 female patients diagnosed at ≥50 y.

All formalin-fixed paraffin-embedded tissues were tested for estrogen (ER) and progesterone receptor (PR) and HER2 expression by immunohistochemistry in tumors derived from the 55 young patients (54 unrelated and one affected sister) and from the 224 women of the additional group (≥50y). HR positive was considered when either ER or PR was positive. FISH analysis was performed to detect *HER2* amplification in tumor samples with a HER2 score 2+ detected by immunohistochemistry reaction. The HER2 status was classified as positive when HER2 (score 3+) was detected by immunohistochemistry or *HER2* DNA amplification was detected by FISH analysis.

## Methods

Full details of methods are given in the online Material and Methods S1.

Briefly, the coding regions including intron-exon boundaries of *BRCA1* (U14680 or NM_007294.3), *BRCA2* (U43746 or NM_000059.1) and *TP53* (NM_000546) genes were sequenced in both the forward and reverse directions, and *CHEK2* (NM_007194.3) was screened for the c.1100delC mutation. Chromatographic tracings were analyzed using the CLC Bio software. Nucleotide alterations were searched in the BIC Database (Breast Information Core; http://research.nhgri.nih.gov/bic, freeze October, 2012). Genes were considered as wild type when the nucleotide missense alterations were classified as no clinical relevance in BIC database and/or as no or little clinical significance (values 1 and 2, respectively) in LOVD-IARC database. Genes were considered as unclassified variant (UV) when the nucleotide missense alterations were categorized as unknown clinical relevance in BIC and/or as uncertain in LOVD-IARC (value 3) database. In cases of disagreement between the two databases, the classification of LOVD-IARC was taken into consideration. Genes with any type of insertion or deletion or amino acid substitution that result in premature_stop codons before amino acids 1853 and 3309 within the BRCA1 and BRCA2, respectively, were classified as mutated. The UVs were submitted to *in silico* prediction programs. Nucleotide ambiguities leading to amino acid changes in the p53 protein were searched in the IARC database (International Agency for Research on Cancer; http://www-p53.iarc.fr/index.html). All detected alterations were confirmed in a second DNA sample in both the forward and reverse directions.

For gene expression analysis, tumor samples of the 55 young patients (54 unrelated patients and one affected sister) were included. One-color labeled cRNAs were hybridized to the Agilent B4X44K G4112F whole human genome oligoarray (Agilent, Santa Clara, USA). Data were analyzed with a permuted t-test (MEV, TM4 software), and genes were considered differentially expressed when p≤0.01 and fold-change ≥|2| (correction by adjusted Bonferroni method). Hierarchical clustering of samples was verified by Pearson correlation distance and complete linkage methods. Over-representation of pathways and biological process in the differentially expressed genes was determined with FunNet software (Functional Analysis of Transcriptional Networks), using KEGG and Gene Ontology (GO) annotations (level 9). All microarray raw data have been deposited in the GEO public database (http://www.ncbi.nlm.nih.gov/geo), a MIAME compliant database, under accession number GSE37126 (http://www.ncbi.nlm.nih.gov/geo/query/acc.cgi?token=bzqzlaugqkeqsle&acc=GSE37126).

Comparative genomic hybridization based on microarrays (array-CGH) was performed for investigating DNA copy number alterations using a 180 K whole-genome platform (Oxford Gene Technology, Oxford, UK) as previously described [25]. Germline array-CGH data were also visually inspected for copy number imbalances within the *BRCA1*, *BRCA2*, and *TP53* genes in resolution of a single probe. The full germline DNA copy number data for the patients without *BRCA1/2* mutations have been previously reported [25].

## Results

### Patient and Tumor Characteristics

For germline mutation screening 54 patients were included in the study (see Table S1 for complete information), with a median age of 31 y (range 22–35 y). Of the 51 unrelated patients interviewed, 16 (31.4%) reported positive familial history [FH(+)].

The majority of all young patients (89%) was diagnosed with invasive ductal carcinoma either of intermediate or high histological grades and early-stage disease (clinical stages I/II, 58%). Most tumors (76.4%) were hormonal receptor-positive [HR(+)] [76.4% ER(+) and 60.0% PR(+)], 20% presented positive HER2 status [HER2(+)] (one patient had unknown HER2 status), and 20% were triple-negative (TN) (complete information in Table S1).

### Analysis of the Hormone Receptor and HER2 Status of Breast Tumors from Early-onset (≤35 y) and Late-onset (≥50 y) Patients

At first, we compared the protein expression of routinely used immunohistochemistry markers [ER/PR for hormonal receptors (HR)] and HER2 status in tumors from the 55 young (≤35 y) (54 unrelated and 1 sister) and old patients (50 y). The latter group comprised 224 patients with a median age of 64 y (50–93 y), all patients presented invasive ductal carcinomas (IDC).

No differences in the frequency of HR(+), HR(−), HER2(+) or TN tumors were detected between early-onset and late-onset breast tumor patient groups (this analysis considered only 49 tumors diagnosed as invasive ductal carcinoma in the group of young patients). Significant differences in high grade and advanced clinical stage frequencies were observed. High-grade tumors were significantly detected in young patients (p = 0.021), while advanced clinical stage tumors occurred more frequently in older patients (p = 0.031) (Table 1).

**Table 1:** Distribution of clinical and histopathological features in young and older patients (considering only IDC histological type).

%	Young patients n = 49	Older patients n = 224	p-value
HG3	36.7	24.7	0.021*
CS III/IV	41.7	58.7	0.031*
HR (+)	79.6	68.6	0.127
HER2 (+)	22.4	12.1	0.157
TN	16.3	21.3	0.437

HG3, High Grade 3; CS, Clinical Stage; HR, hormonal receptor; TN; Triple Negative.

(*)Statistically significant.

### Frequency of *BRCA1*, *BRCA2*, *TP53* and *CHEK2 (c.1100delC)* Mutations in Brazilian Patients ≤35 y

Deleterious mutations in *BRCA1* and *BRCA2* genes were found in 11 of the 54 (20.5%) of the unrelated patients. Thirty-two patients were classified as *BRCA1/2* wild type (59%) and 10 as UV carriers (18.5%). Mutations were detected in 7 (13%) patients for *BRCA1* gene and in 4 (7.5%) patients for *BRCA2* (Table 2).

**Table 2:** Deleterious mutations detected in the *BRCA1*, *BRCA2* and *TP53* genes.

Patients	Age at diagnosis	Familial History	Gene	Alteration	Reference	Type	Description	HR	HER2
ID_1014	29	(+)	*BRCA1*	c.560+2T>A		IVS	current study	pos	neg
ID_2017	29	(+)	*BRCA1*	c.5382insC	[49], [50] ^a^	Frameshift	BIC	neg	neg
ID_2021	27	(+)	*BRCA1*	c.300T>G - p.C61G		Missense	BIC	neg	neg
ID_2023	33	(+)	*BRCA1*	c.5382insC	[49], [50] ^a^	Frameshift	BIC	neg	pos
ID_2025	35	(−)	*BRCA2*	c.3034del4		Frameshift	BIC	pos	neg
ID_2026	31	(−)	*BRCA1*	c.3450del4	[49] ^a^	Frameshift	BIC	neg	neg
ID_2031	24	(+)	*BRCA2*	c.2494C>T - p.Q756X		Nonsense	current study	pos	neg
ID_2032	29	ND	*BRCA2*	c.4968insGT		Frameshift	current study	pos	neg
ID_2034	25	(+)	*BRCA1*	c.5370C>T - p.R1751X		Nonsense	BIC	neg	neg
ID_2039	24	(−)	*TP53*	c.427G>A - p.V143M		Missense	IARC	pos	neg
ID_2048	35	(−)	*BRCA2*	c.5190T>A - p.C1654X		Nonsense	current study	pos	neg
ID_4010	35	(+)	*BRCA1*	c.2524delTG		Frameshift	BIC	neg	neg

BIC, Breast Cancer Information Core; IARC, International Agency for Research on Cancer; HR, hormonal receptor status; (a), mutation identified in Brazilian patients reported by others; ND: not determined – (ID_2032 patient is adopted).

Three of these *BRCA2* mutations and one of *BRCA1* were reported for the first time [*BRCA2:* p.Q756X, p.C1654X and c.4968insGT that results in a premature stop codon at amino acid 1617; *BRCA1*: c.560+2T>A, a splice-site variant that leads in an aberrant transcript with a premature stop codon (data not shown)].

In the 10 UV-carrier patients, 8 distinct missense alterations were identified (p.T1915M detected in two unrelated patients and p.I2490T in four unrelated patients) (Table 3). Three UVs have not been previously described (p.S1655P and p.A1669V in *BRCA1* and p.D381G in *BRCA2*).

**Table 3:** Unclassified Variants (UVs) identified in *BRCA1* and *BRCA2* genes.

DNA change	protein change	N	Gene	Exon	BIC	LOVD-IARC	Polyphen	SIFT	Align GVGD
c.5082T>C	p.S1655P	1	*BRCA1*	16	not described	no result	Possibly damaging	Tolerated	C65
c.5125C>T	p.A1669V	1	*BRCA1*	17	not described	no result	Possibly damaging	Affect	C0
c.1370A>G	p.D381G	1	*BRCA2*	11	not described	no result	Benign	Tolerated	C0
c.5972C>T	p.T1915M	2	*BRCA2*	11	unknown	no result	Possibly damaging	Tolerated	C0
c.6550C>T	p.R2108C	1	*BRCA2*	11	unknown	no result	Probably damaging	Tolerated	C0
c.7697T>C	p.I2490T	4	*BRCA2*	15	unknown	no result	Possibly damaging	Tolerated	C45
c.9058A>T	p.I2944F	1	*BRCA2*	22	unknown	no result	Possibly damaging	Affect	C0
c.10462A>G	p.I3412V	1	*BRCA2*	27	unknown	no result	Benign	Tolerated	C0

N, number of probands who harbor the UV; Exon: where the UV is mapped; BIC, Breast Cancer Information Core (not described in BIC database; unkown: with unknown clinical relevance); LOVD-IARC (no result: not classified in LOVD-IARC database); Align GVGD, C0, less likely to interfere in protein function; C15, C45, C55, C65, more likely to interfere in protein function.

The 1100-deletion in the *CHEK2* gene was not found in any of the samples studied. Finally, 43 patients negative for *BRCA1/2* pathogenic mutations were also screened for *TP53*, and only one was found to be mutated. This pathogenic alteration (p.V143M) has already been reported in a tumor as a somatic mutation in the IARC database.

### Relationship between Mutation Status and Tumor Subtype and Familial History of Hereditary Cancer

Positive significant associations were observed between *BRCA1/2*-mutated carriers with both HR(−) and triple-negative (TN) tumors. No significant association was found between *BRCA1/2*-mutated carriers and HER2 status of tumors (Table 4).

**Table 4:** Distribution of *BRCA1/2* status according to immunohistochemical characteristics and familial history.

	*BRCA1/2 WT n (%)*	*BRCA1/2 UV n (%)*	*BRCA1/2 MUT n (%)*	
FH(+); n = 16	5 (31.3)	4 (25.0)	7 (43.7)	p = 0.006*
FH(−); n = 35	25 (71.4)	7 (20)	3 (8.6)	
HR(+); n = 42	27 (64.3)	10 (23.8)	5 (11.9)	p = 0.026*
HR(−); n = 13	5 (38.5)	2 (15.4)	6 (46.1)	
TN; n = 11	4 (36.4)	2 (18.2)	5 (45.4)	p = 0.059
NTN; n = 44	28 (63.6)	10 (22.7)	6 (13.6)	
HER2(+) n = 11	9 (81.8)	1 (9.1)	1 (9.1)	p = 0.185
HER2(−) n = 43	22 (51.2)	11 (25.6)	10 (23.2)	

WT, wild type; UV, unclassifed variant; MUT, mutated; FH, cancer family history; HR, hormonal receptor tumor; TN, triple negative; NTN, non-triple negative; (+), positive; (−), negative; p, Pearson chi-square.

(*) Statistically significant with a 95% confidence interval; for familial history distribution the 54 unrelated young patients were considered; for distribution of HR, TN/NTN and HER2 status the 55 young patients (54 unrelated and one sister) were considered.

Patients reporting FH(+) had a significant higher probability of harboring *BRCA1/2* mutation. Of the 10 unrelated_patients carrying *BRCA1/2* mutations (7 in *BRCA1* and 3 in *BRCA2*) for whom family history was known, 7 reported a positive familial history (70%). Of the 16 unrelated patients with FH(+), 37.5% carried pathogenic germline mutations in *BRCA1* gene, against only 6.2% in *BRCA2*, revealing that FH(+) is one of the major risk factor for *BRCA1* mutations in Brazilian young patients.

By evaluating the frequency of tumor subtypes as a function of the mutation status of individual genes, our data revealed that 6 out of 7 (85.7%) *BRCA1* mutation-carriers developed HR(−) tumors; among them, 5 were TN (71.4%). All 5 patients who were *BRCA2* or *TP53* mutation-carriers developed HR(+) tumors.

Of the young unrelated patients with HR(−) tumors, 50% (6/12) harbored a deleterious germline mutation in the *BRCA1* gene; this frequency was similar (5/10) in patients with TN tumors. In contrast, in unrelated patients diagnosed with HR(+) tumors, only 9.5% (4/42) and 2.4% (1/42) harbored a deleterious germline mutation in *BRCA2* and *TP53*, respectively.

Finally, analysis of both tumor subtype and FH(+) revealed that 83% (5/6) and 80% (4/5) of patients with FH(+) diagnosed with HR(−) or TN tumors carried a *BRCA1* mutation, respectively. No association was observed among patients with FH(+) diagnosed with HR(+) tumor subtype and mutations in *BRCA2* or *TP53* genes*;* of 10 patients, only one was a *BRCA2* carrier (10%). The *TP53*-mutation carrier was a 24 y patient who did not report a family history of cancer.

### Germline and Tumor Genomic Imbalances in Early-onset Breast Cancer Patients

We also assessed germline and somatic genomic imbalances in 15 patients (blood and tumor matched samples), 7 of which carried *BRCA1/2* germline mutations (3 in *BRCA1* and 4 in *BRCA2*), and 6 and 2 of which harbored *BRCA1/2* wild type and *BRCA1/2* UVs, respectively. This analysis had two basic purposes: first, to search for germline intragenic deletions and/or duplications in *BRCA1, BRCA2*, *TP53* and *CHEK2* genes; and second, to compare the number of somatic copy number alterations (SCNA) in tumors driven or not driven by *BRCA1*/*2* germline mutations.

No germline deletions or duplications were observed in the four genes by array-CGH analysis. Additionally, *BRCA1/2*-mutated tumors did not exhibit a higher degree of genomic instability relative to wild-type- and UV-associated tumors, at least as measured by the total number of SCNAs (Table S2). However, we observed that 3 out of 4 tumors associated with germline *BRCA2* mutations exhibited deletion of the *BRCA2* gene in mosaic (IDs 2048, 2031 and 2025).

### Gene Expression Analysis of Breast Tumors: Identification of Differentially Expressed Genes in *BRCA1/2*-associated and −negative Tumors

To identify a differential gene signature associated to *BRCA1/2* deleterious mutation gene expression analysis was performed in 49 samples for which the tumors were available (10 tumors from *BRCA1/2* mutated carriers; 28 from wild-type *BRCA1/2* patients; 10 from *BRCA1/2* UV-carriers, and 01 from *TP53*-mutated carrier). For this analysis, gene expression profile of 28 tumors from wild-type *BRCA1/2* patients was compared with the 10 tumors from *BRCA1/2*-mutated carriers.

This analysis revealed 34 differentially expressed genes: 18 up-regulated in *BRCA1/2*-mutated tumors and 16 up-regulated in *BRCA1/2*-negative tumors (Table 5). To provide functional insights, we annotated these 34 genes in the biological process category of the Gene Ontology (GO) and KEGG pathways. Thirty-one genes could be categorized in GO (Tables S3 and S4). The over-represented Biological Process (GO) categories for genes up-regulated in the mutated tumors included mainly DNA damage, cellular and cell cycle-related processes. In contrast, the up-regulated genes in negative tumors were preferentially included in cell and embryo development-related processes (Figure S1). Within the KEGG categories, up-regulated genes in *BRCA1/2*-associated tumors were enriched for cell cycle pathways, mismatch repair, glutathione metabolism, oocyte meiosis and progesterone-mediated oocyte maturation. None of the KEGG categories were enriched in the group of genes up-regulated in *BRCA1/2*-negative tumors.

**Table 5:** Differentially expressed genes between BRCA1/BRCA2-negative and -positive mutation-driven tumors.

Genes up-regulated in *BRCA1/BRCA2*-associated tumors				Genes up-regulated in *BRCA1/BRCA2*-negative tumors			
Gene Symbol	Fold	Gene Symbol	Fold	Gene Symbol	Fold	Gene Symbol	Fold
*UBE2E3*	2.0	*BUB1*	2.4	*HTR7*	2.0	FRY*	2.4
*E2F7*	2.0	*ASPM*	2.4	TRAF3IP1*	2.0	*OVGP1*	2.5
*SIN3B*	2.0	*PLK1*	2.4	*TMEM135*	2.0	*SLC16A5*	2.8
*RRM2*	2.1	*EXO1*	2.4	*YPEL2*	2.1	*KIF9*	2.8
FMR1*	2.2	*CENPN*	2.6	*SMARCE1*	2.1	*TBX3*	2.8
TMPO*	2.2	*MELK*	2.6	*RABEP1*	2.2	ZNF396*	3.0
*CDCA8*	2.2	*HMGB3P1*	3.1	IFT140*	2.2	TCAP*	3.1
*UBE2T*	2.3			*TPTE2P5*	2.3	*TC1*	3.2
*CDCA3*	2.3			*USP35*	2.3	SRCIN1*	4.4

(*)Concordant results in gene expression and array-CGH analysis.

**Figure 1:**
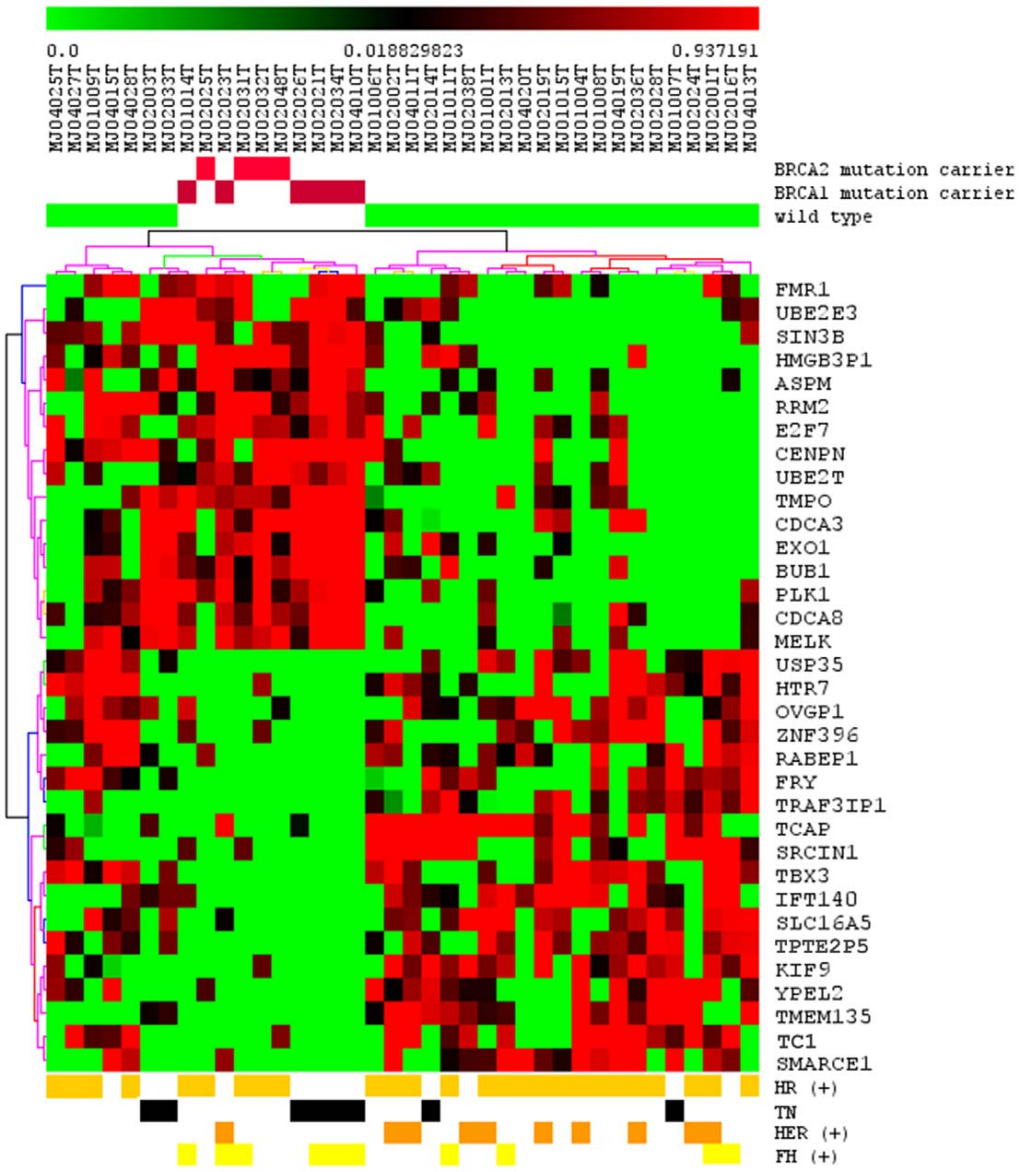
Hierarchical clustering based on 34 differentially expressed genes in *BRCA1/BRCA2*-associated and -negative tumors. Each row represents a gene, and each column represents a tumor sample. Red indicates strong expression; green indicates weak expression; and black indicates moderate expression. Red squares represent *BRCA1* or *BRCA2* pathogenic-associated tumors, and green squares represent tumors from *BRCA1/2* WT (non mutated). The colored lines of the dendrogram represent the support for each clustering: black and gray lines indicate greater reliability; yellow and red lines indicate lesser reliability.

Hierarchical clustering based on this set of genes discriminated 100% of the *BRCA1/2*-associated tumors from 75% of the *BRCA1/2*-negative tumors (Figure 1).

Next, we performed hierarchical clustering including the additional 10 tumors from patients carrying UVs in the *BRCA1*/*2* genes and the *TP-53* associated tumor (Figure S2). Interestingly, the clustering based on gene expression of the 49 tumor samples grouped 93% of the *BRCA1/2*-negative tumors discriminating from 100% of *BRCA1/2*-associated tumors. In regarding to UV breast tumor samples, 3 (30%) and 7 (70%) out of 10 samples were clustered with *BRCA1/2*-associated and -negative tumors, respectively. Tumors from two affected sisters (IDs 2007 and 2012) whose germline UV identified in the index patient (ID 2007) was confirmed in the sister (ID 2012) (*BRCA1*- p.S1655P) were discriminated into two different cluster ramifications; these two tumors were of different subtypes: one was TN, high-grade and atypical medullar, while the other was invasive ductal carcinoma, ER(+), HER(−) and of histological grade 2. The *TP53*-associated tumor clustered with the *BRCA1/2*-associated tumor group.

### Combined Analysis of Gene Expression and Chromosomal Imbalances of Breast Tumors

The set of 34 genes identified as up- or down-regulated in the group of *BRCA1/2*-associated tumors was interrogated for DNA gains and losses. We considered a concordant pattern when at least two tumors in each group exhibited: a) gains for up-regulated genes in *BRCA1/2*-associated tumors and/or loss in -negative tumors, b) losses for down-regulated genes in *BRCA1/2*-associated tumors and/or gains in -negative tumors. A total of 8 genes displayed a concordant pattern namely *FMR1* and *TMPO* (up-regulated), and *SRCIN1*, *TCAP*, *ZNF396*, *IFT140*, *FRY* and *TRAF3IP1* (down-regulated) (Table 5). A discordant opposite pattern was not observed. The remaining genes were either not affected by SCNA or were randomly affected by gains and losses irrespective to the *BRCA1/2* mutational status.

## Discussion

An increased risk of death has been observed in young women affected by breast cancer [9], implying that tumors in early-onset cancer patients could be a distinct entity of breast cancer. Tumor aggressiveness in young women has been reported worldwide based on increases in the rate of high-grade, fast-proliferation, HR(−), basal-like and HER2-enriched breast tumors [1]–[3], [9], [13], [15], [26], [27] and their poorer overall and disease-free survival rates [5]. In the current study, tumor aggressiveness was assessed by comparing groups of tumors from younger and older women. Our results revealed a higher percentage of high histological tumor grade in the early-onset breast cancer group compared with the late-onset group, similar to other studies [4]. We did not detect an increase of HR(−) or TN breast tumors in young women, finding in contrast to those previously reported in the Brazilian [28]–[30] and other populations [1], [12] and in agreement with some studies [3], [4].

Inactivating mutations in cancer susceptibility genes, such as *BRCA1* and *BRCA2,* which are inherited in an autosomal dominant pattern, are the major genetic factor associated with a high risk of breast cancer at an early age. The percentage of *BRCA1/2* germline mutations in early-onset breast cancer patients ranges widely, from 11 to 24% in different studies [16], [18], [31]–[33]. Here, we reported a 20.4% rate of *BRCA1/2* deleterious mutation, a frequency comparable to those described in Caucasian, Korean, American [<36 y (16.7%)] [18], British [<31 y (16%)] [16], Canadian [<36 y (16%)] [32] and Cypriot patients [<40 y (23%)] [33] and distinct to French patients [<36 y (10.9%)] [34].

Our data pointed out that *BRCA1* mutation screening is mandatory for young Brazilian patients diagnosed with HR(−) and/or TN breast tumors, specially when it occurs in combination with FH(+), supporting previous studies that have reported an increased probability of *BRCA1* germline mutation in_young patients with FH(+) and TN tumors [35]–[37]. It is well known that Brazilian population harbor a complex genetic background, reinforcing that both features, negative hormonal receptor tumors [HR(−) and/or TN] and FH(+), are very solid risk factors for *BRCA1* mutation in young women, irrespective of their genetic composition. Nevertheless, an extensive evaluation of the prevalence of the *BRCA1* mutation in TN and HR(−) tumors similar to that performed in the British population [37] is needed for proper genetic counseling of individuals and families at higher risk of breast cancer in Brazil.

Eighteen and a half percent of our patients (10 out of 54) presented *BRCA1/2* UVs, and most of these patients were diagnosed with HR(+) tumors. The reported frequencies of *BRCA* UVs vary in different ethnic populations, with higher rates in African-American (38%) than in Caucasian (10%) and Korean patients (12%) [18]. The intermediary UV frequency in the patients in our study (18.5%) may reflect the high genetic miscegenation of the Brazilian population.

Among the eight types of UVs found in this study, the variant *BRCA2:* c.7697T>C, p.I2490T was detected in four distinct young patients, one of them is a carrier of a novel nonsense *BRCA2* mutation (c.5190T>A - p.C1654X). This fact suggests low likelihood of this variant to play a deleterious function and consequently to be a disease-causing mutation.

Another important genetic factor related to early-onset breast cancer is the occurrence of germline *TP53* mutations, which are associated with Li-Fraumeni Syndrome or Li-Fraumeni-like syndromes. In this cohort, a germline *TP53* mutation was detected in only one case, in line with others studies that found very low frequencies of *TP53* mutations (1%) in early-onset patients [38], [39]. Although the *TP53* pR337H mutation was reported as a founder effect mutation in the population of southern Brazil [39] and has been detected at high frequency in Brazilian families with high cancer predisposition [40], we did not detected this specific mutation. This result can be attributed to the relatively low penetrance of this mutation for breast cancer in women below the age of 30 y [39].

No germline copy number alterations affecting the *BRCA1*, *BRCA2, TP53 or CHEK2* genes were identified. A whole-genome investigation in Brazilian early-onset and FH(+) breast cancer patients detected rare germline CNVs [25]; one of the reported patients and her affected sister carried a 540 kb 1p31.1 microdeletion encompassing only 3 genes (*ST6GALNAC3, ST6GALNAC5, PIGK*); both patients were included in the present study. The most relevant gene in the affected region is *ST6GALNAC5*, a sialyltransferase recently identified as related to the development of breast cancer metastasis [41], suggesting a possible role for this gene in the development of the early-onset breast cancer in these patients.

Gene expression signatures have also been used for distinguishing breast tumor subtypes [14], chemotherapy-resistant and -sensitive samples [42], and pre-invasive lesions with distinct malignant potential [43], demonstrating that it is a very efficient approach for categorizing heterogeneous tumors. In the current study, we identified a transcriptional signature associated with *BRCA1/2* status that distinguished *BRCA1/2*-associated tumors from negative tumors and suggested distinct biological processes involved in driving transformation in these tumor groups of young patients. The intrinsic molecular subtypes determined by gene expression profile strongly influence patient prognosis [44] and surely other important tumor characteristics. Three genes (*RRM2*, *UBE2T* and *EXO1*) belonged to the list of 50 genes associated to molecular subtype (PAM50) [45] were detected in the gene expression signature associated to *BRCA1/2* status. Therefore, if the gene expression modulation of these three genes is really influenced by *BRCA1/2* mutations or by the molecular subtypes is hard to be estimated.

Interestingly, 3 of the 8 genes exhibiting a concordant pattern in the genomic and transcriptional analysis (*FMR1, SRCIN1* and *TCAP)*_are annotated in those over-represented categories, reinforcing the involvement of defective cellular- and embryo development-related processes in triggering breast tumorigenesis in *BRCA1/2*-associated and -negative groups, respectively. *FMR1,* up-regulated in *BRCA1/2*-associated tumors, is located in chromosome Xq27.3. The protein encoded by *FMR1* binds RNA and seems to be involved in the traffic of mRNAs from the nucleus to the cytoplasm. Remarkably, mutation in this gene has been associated with ovarian cancer risk [44]. Both *SRCIN1* and *TCAP* genes, up-regulated in *BRCA1/2*-negative tumors, are located in 17q12. SRCIN1 protein, also known as p140CAP, regulates the oncogene SRC kinase interfering in balance from SRC active to inactive [46].

p140CAP arrests E-cadherin at the cell membrane and prevents EGFR and Erk1/2 signaling, decreasing proliferation of tumor cells [47]. The protein encoded by *TCAP*_is found in striated and cardiac muscle and mutation in this gene has been associated with limb-girdle muscular dystrophy type 2G [48]. Although this gene is mapped in a region commonly amplified in breast tumor, nothing is known about its role in the tumor context. All three genes are promising candidates that deserve further investigation of their role in breast cancer, especially in the context of *BRCA1/2* status.

The experimental approach, combining germline and somatic analysis, has shed light on some of the genetic factors that trigger the development of breast cancer at an early age, which will aid in establishing additional criteria for genetic testing. Altogether, data delineated an initial portrait of Brazilian early-onset breast cancer patients, contributing to the establishment of public health standards for referring patients for genetic testing and leading to more personalized and effective management of breast cancer in Brazil.

## Supporting Information

Figure S1 **GO biological process-enriched categories of the up- and down-regulated genes in** ***BRCA1/2-*** **associated tumors**. The bar corresponds to the percentage of differentially expressed genes in relation to all annotated genes in the respective category.
(TIF)

Figure S2 **Hierarchical clustering based on 34 differentially expressed genes in** ***BRCA1/BRCA2*** **-associated and -negative tumors.** Each row represents a single gene, and each column represents a tumor sample. Red indicates strong expression; green indicates weak expression; and black indicates moderate expression. Red squares represent *BRCA1* or *BRCA2* pathogenic-associated tumors, and green and blue squares represent tumors from *BRCA1/2* non-mutated and unclassified variant carriers, respectively. Purple square represents tumor from *TP53* mutated carrier. The colored lines of the dendrogram represent the support for each clustering: black and gray lines indicate greater reliability; yellow and red lines indicate lesser reliability.
(TIF)

Table S1 Clinical data of the patients included in the study.
(DOC)

Table S2 Summary of the array-CGH results of fifteen tumor samples.
(DOC)

Table S3 Up-regulated genes in *BRCA1/2*-associated and -negative tumors in the enriched GO Biological Process categories.
(DOC)

Table S4 Up-regulated genes in *BRCA1/2*-associated tumors distributed in the enriched categories of the KEGG pathway.
(DOC)

Material and Methods S1 **Full details of methods.**
(DOC)
