# Supplementary figures and images for: Comprehensive Analysis of BRCA1, BRCA2 and TP53 Germline Mutation and Tumor Characterization: A Portrait of Early-Onset Breast Cancer in Brazil
Source: PLoS One. 2013 Mar 1;8(3):e57581. doi: 10.1371/journal.pone.0057581 (PMC3586086; doi:10.1371/journal.pone.0057581)

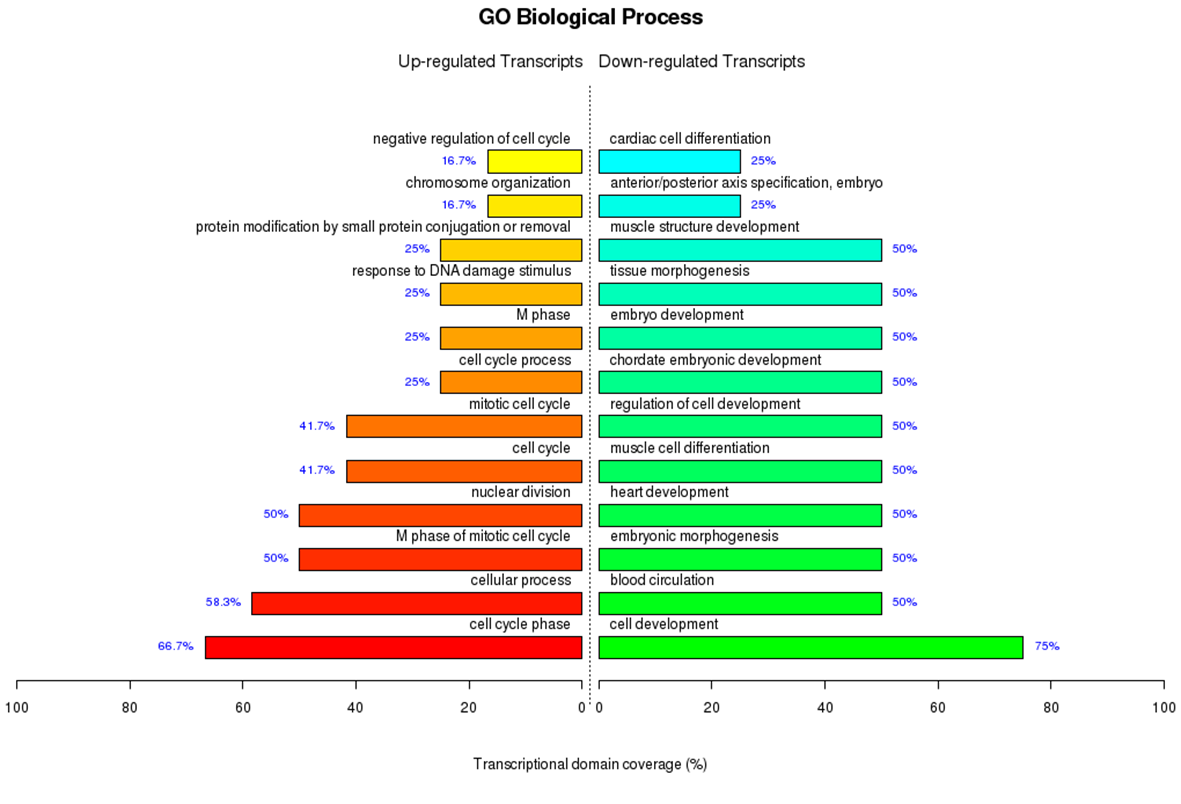

Supplement: Figure S1 — GO biological process-enriched categories of the up- and down-regulated genes in BRCA1/2- associated tumors. The bar corresponds to the percentage of differentially expressed genes in relation to all annotated genes in the respective category. (TIF) [file pone.0057581.s001.tif]

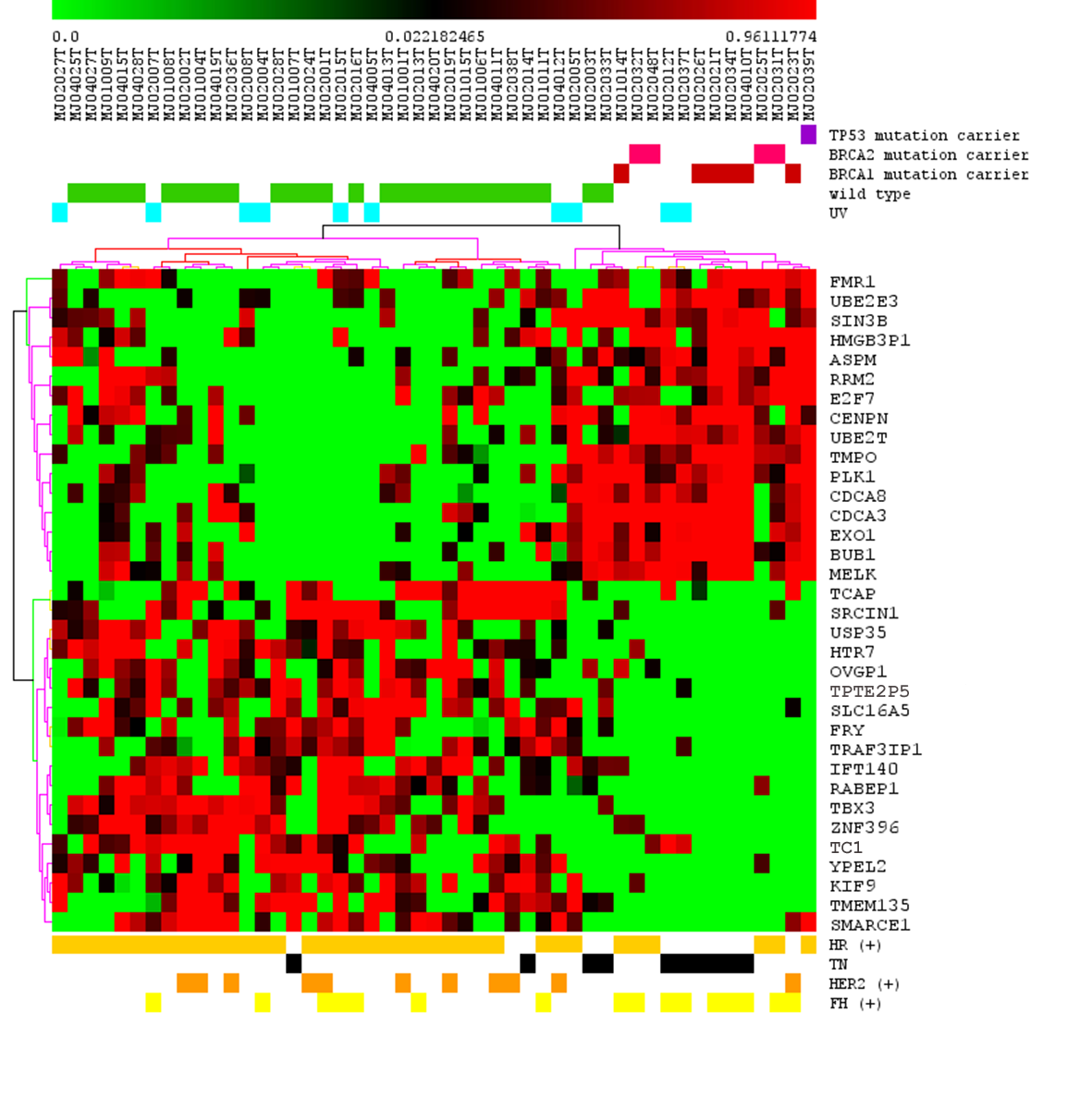

Supplement: Figure S2 — Hierarchical clustering based on 34 differentially expressed genes in BRCA1/BRCA2 -associated and -negative tumors. Each row represents a single gene, and each column represents a tumor sample. Red indicates strong expression; green indicates weak expression; and black indicates moderate expression. Red squares represent BRCA1 or BRCA2 pathogenic-associated tumors, and green and blue squares represent tumors from BRCA1/2 non-mutated and unclassified variant carriers, respectively. Purple square represents tumor from TP53 mutated carrier. The colored lines of the dendrogram represent the support for each clustering: black and gray lines indicate greater reliability; yellow and red lines indicate lesser reliability. (TIF) [file pone.0057581.s002.tif]
