# Supplementary material for: Comprehensive Analysis of BRCA1, BRCA2 and TP53 Germline Mutation and Tumor Characterization: A Portrait of Early-Onset Breast Cancer in Brazil
Source: PLoS One. 2013 Mar 1;8(3):e57581. doi: 10.1371/journal.pone.0057581 (PMC3586086; doi:10.1371/journal.pone.0057581)
Supplement: Table S1 — Clinical data of the patients included in the study. (DOC) [file pone.0057581.s003.doc]

**Table S1. Clinical data of the patients included in the study.**

| **Patient** | **Age at diagnosis** | **FH** | ***BRCA1*, *BRCA2* and *TP53* status** | **Mutation description** | **UV description** | **V description** | **CS** | **HT** | **HG** | **ER** | **PR** | **HR** | **HER2 Status** | **Tumor sub-classification** | **Microarray** |
| --- | --- | --- | --- | --- | --- | --- | --- | --- | --- | --- | --- | --- | --- | --- | --- |
| ID_1001 | 33 | (-) | Wild Type | *-* | - | - | 2 | IDC | 3 | pos | pos | pos | pos | HR (+) | yes |
| ID_1004 | 34 | (-) | Wild Type | *-* | - | - | 2 | IDC | 2 | pos | pos | pos | pos | HR (+) | yes |
| ID_1006 | 32 | (-) | Wild Type | *-* | *-* | *BR1: c.4158A>G, p.R1347G; BR2: c.6328C>T, p.R2034C* | 2 | IDC | 3 | pos | pos | pos | ND | HR (+) | yes |
| ID_1007 | 25 | (-) | Wild Type | *-* | *-* | *BR1: c.3238G>A, p.S1040N* | 4 | IDC | 2 | neg | neg | neg | neg | HR (-) / TN | yes |
| ID_1008 | 33 | (-) | Wild Type | *-* | - | - | 1 | IDC | 2 | pos | neg | pos | neg | HR (+) | yes |
| ID_1009 | 33 | (-) | Wild Type | *-* | - | - | 2 | IDC | 2 | pos | neg | pos | neg | HR (+) | yes |
| ID_1010 | 31 | ND | Wild Type | *-* | - | - | 2 | IDC | 2 | pos | pos | pos | neg | HR (+) | no |
| ID_1011 | 33 | (+) | Wild Type | *-* | *-* | *BR1: c.3238G>A, p.S1040N* | 1 | ILC | No | pos | pos | pos | neg | HR (+) | yes |
| ID_1012 | 30 | ND | Wild Type | *-* | - |  | ND | IDC | 2 | pos | neg | pos | neg | HR (+) | no |
| ID_1014 | 29 | (+) | MUT | *BR1: c.560+2T>A* | - | - | 1 | ILC | No | pos | pos | pos | neg | HR (+) | yes |
| ID_1015 | 33 | (-) | Wild Type | *-* | - | - | 2 | PAP | No | pos | neg | pos | neg | HR (+) | yes |
| ID_2001 | 28 | (+) | Wild Type | *-* | *-* | *BR1: c.1186A>G, p.Q356R* | 2 | IDC | 2 | pos | pos | pos | pos | HR (+) | yes |
| ID_2002 | 30 | (-) | Wild Type | *-* | - |  | 2 | IDC | 2 | pos | pos | pos | pos | HR (+) | yes |
| ID_2003 | 26 | (-) | Wild Type | *-* | *-* | *BR1: c.1186A>G, p.Q356R* | 2 | IDC | 3 | neg | neg | neg | neg | HR (-) / TN | yes |
| ID_2004 | 34 | (+) | UV | *-* | *BR2: c.5972C>T, p.T1915M* | *-* | 2 | IDC | 2 | pos | pos | pos | neg | HR (+) | yes |
| ID_2005 | 29 | (-) | UV | *-* | *BR2: c.6550C>T, p.R2108C ; c.7697T>C, p.I2490T* | *-* | 2 | IDC | 3 | pos | neg | pos | neg | HR (+) | yes |
| ID_2006 | 35 | (+) | Wild Type | *-* | - | - | 2 | IDC | 2 | pos | pos | pos | neg | HR (+) | no |
| ID_2007* | 29 | (+) | UV | *-* | *BR1: c.5082T>C, p.S1655P* | *-* | 3 | IDC | 3 | pos | pos | pos | neg | HR (+) | yes |
| ID_2008 | 33 | (-) | UV | *-* | *BR2: c.9058A>T, p.I2944F* | *-* | 3 | IDC | 3 | pos | neg | pos | neg | HR (+) | yes |
| ID_2012* | 29 | (+) | UV | *-* | *BR1: c.5082T>C, p.S1655P* | *-* | 1 | IDC | 3 | neg | neg | neg | neg | HR (-) / TN | yes |
| ID_2013 | 29 | (+) | Wild Type | *-* | - | - | 2 | IDC | 3 | pos | pos | pos | neg | HR (+) | yes |
| ID_2014 | 35 | (-) | Wild Type | *-* | - | - | 2 | MED | 3 | neg | neg | neg | neg | HR (-) / TN | yes |
| ID_2015 | 34 | (+) | UV | *-* | *BR2: c.1370A>G, p.D381G* | *BR1: c.1186A>G, p.Q356R* | 3 | IDC | 2 | pos | pos | pos | neg | HR (+) | yes |
| ID_2016 | 35 | (+) | Wild Type | *-* | - | - | 1 | IDC | 2 | pos | pos | pos | neg | HR (+) | yes |
|  |  |  |  |  |  |  |  |  |  |  |  |  |  |  |  |
| **Patient** | **Age at diagnosis** | **FH** | ***BRCA1*, *BRCA2* and *TP53* status** | **Mutation description** | **UV description** | **V description** | **CS** | **HT** | **HG** | **ER** | **PR** | **HR** | **HER2 Status** | **Tumor sub-classification** | **Microarray** |
| ID_2017 | 29 | (+) | MUT | *BR1: c.5382insC* | - | - | 2 | IDC | 3 | neg | neg | neg | neg | HR (-) / TN | No |
| ID_2019 | 34 | (-) | Wild Type | *-* | - | - | 4 | IDC | 1 | pos | neg | pos | pos | HR (+) | yes |
| ID_2021 | 27 | (+) | MUT | *BR1: c.300T>G - p.C61G* | - | *BR1: c.1186A>G, p.Q356R* | TX | MET | ND | neg | neg | neg | neg | HR (-) / TN | yes |
| ID_2023 | 33 | (+) | MUT | *BR1: c.5382insC* | - | *BR1: c.2640C>T, p.R841W; c.3238G>A, p.S1040N; c.5236G>C, p.G1706A* | 2 | IDC | 3 | neg | neg | neg | pos | HR (-) | yes |
| ID_2024 | 35 | (-) | Wild Type | *-* | - | - | 2 | IDC | 3 | pos | pos | pos | pos | HR (+) | yes |
| ID_2025 | 35 | (-) | MUT | *BR2: c.3034del4* | - | - | 2 | IDC | 3 | pos | pos | pos | neg | HR (+) | yes |
| ID_2026 | 31 | (-) | MUT | *BR1: c.3450del4* | - | - | 2 | MED | ND | neg | neg | neg | neg | HR (-) / TN | yes |
| ID_2027 | 35 | (-) | UV | *-* | *BR2: c.7697T>C, p.I2490T* | *-* | 3 | IDC | 2 | pos | pos | pos | neg | HR (+) | yes |
| ID_2028 | 32 | (-) | Wild Type | *-* | - | - | 2 | IDC | 2 | pos | pos | pos | neg | HR (+) | yes |
| ID_2031 | 24 | (+) | MUT | *BR2: c.2494C>T - p.Q756X* | - | - | 4 | IDC | 2 | pos | pos | pos | neg | HR (+) | yes |
| ID_2032 | 29 | adopted | MUT | *BR2: c.4968insGT* | - | - | 3 | IDC | 3 | pos | pos | pos | neg | HR (+) | yes |
| ID_2033 | 34 | (-) | Wild Type | *-* | - | - | 2 | IDC | 2 | neg | neg | neg | neg | HR (-) / TN | yes |
| ID_2034 | 25 | (+) | MUT | *BR1: c.5370C>T - p.R1751X* | - | - | 2 | IDC | 3 | neg | neg | neg | neg | HR (-) / TN | yes |
| ID_2036 | 26 | (-) | Wild Type |  | - | - | 4 | IDC | 2 | pos | pos | pos | pos | HR (+) | yes |
| ID_2037 | 28 | (+) | UV | *-* | *BR1: c.5125C>T, p.A1669V* | *-* | 3 | IDC | 3 | neg | neg | neg | neg | HR (-) / TN | yes |
| ID_2038 | 22 | (-) | Wild Type | *-* | - | - | 2 | IDC (PAGET) | 2 | neg | neg | neg | pos | HR (-) | yes |
| ID_2039 | 24 | (-) | MUT | *TP53: c.427G>A - p.V143M* | *-* | *BR1: c.1186A>G, p.Q356R* | 3 | IDC | 3 | pos | pos | pos | neg | HR (+) | yes |
| ID_2048 | 35 | (-) | MUT | *BR2: c.5190T>A - p.C1654X* | *BR2: c.7697T>C, p.I2490T* | *BR2: c.7378C>A, p.Q2384K; c.9463G>A, p.V3079I* | 3 | IDC | 3 | pos | neg | pos | neg | HR (+) | yes |
| ID_4005 | 29 | (-) | UV | *-* | *BR2: c.7697T>C, p.I2490T* | *-* | 3 | IDC | 2 | pos | pos | pos | neg | HR (+) | yes |
| ID_4010 | 35 | (+) | MUT | *BR1: c.2524delTG* | - | - | 2 | IDC | 3 | neg | neg | neg | neg | HR (-) / TN | yes |
| **Patient** | **Age at diagnosis** | **FH** | ***BRCA1*, *BRCA2* and *TP53* status** | **Mutation description** | **UV description** | **V description** | **CS** | **HT** | **HG** | **ER** | **PR** | **HR** | **HER2 Status** | **Tumor sub-classification** | **Microarray** |
| ID_4011 | 29 | (-) | Wild Type | *-* | *-* | *BR2: c.6448C>A, p.H2074N* | 1 | IDC | 2 | pos | pos | pos | pos | HR (+) | yes |
| ID_4012 | 27 | (-) | UV | *-* | *BR2: c.10462A>G, p.I3412V* | *-* | 4 | IDC | 2 | pos | pos | pos | pos | HR (+) | yes |
| ID_4013 | 29 | (-) | Wild Type | *-* | *-* | *-* | 1 | IDC | 2 | pos | pos | pos | neg | HR (+) | yes |
| ID_4015 | 31 | (-) | Wild Type | *-* | *-* | *-* | 3 | IDC | 2 | pos | pos | pos | neg | HR (+) | yes |
| ID_4016 | 32 | (-) | UV | *-* | *BR2: c.5972C>T, p.T1915M* | *BR1: c.1186A>G, p.Q356R* | 3 | IDC | 2 | pos | neg | pos | neg | HR (+) | no |
| ID_4019 | 33 | (-) | Wild Type | *-* | *-* | *-* | 2 | IDC | 1 | pos | pos | pos | neg | HR (+) | yes |
| ID_4020 | 35 | (-) | Wild Type | *-* | *-* | *-* | 3 | IDC | 2 | pos | pos | pos | neg | HR (+) | yes |
| ID_4022 | 34 | (-) | Wild Type | *-* | *-* | *BR1: c.1186A>G, p.Q356R* | 4 | IDC | 2 | pos | pos | pos | neg | HR (+) | no |
| ID_4025 | 30 | (-) | Wild Type | *-* | *-* | *-* | 3 | IDC | 2 | pos | pos | pos | neg | HR (+) | yes |
| ID_4027 | 34 | (-) | Wild Type | *-* | *-* | *-* | 3 | IDC | 2 | pos | pos | pos | neg | HR (+) | yes |
| ID_4028 | 28 | (-) | Wild Type | *-* | *-* | *-* | 2 | IDC | 2 | pos | pos | pos | neg | HR (+) | yes |

ID, patient identity; age in years at surgery; FH, familial history; UVs, unclassified variants; MUT, mutated; CS, clinical stage; HT, histological type; IDC, invasive ductal carcinoma; ILC, invasive lobular carcinoma; MED, medullary carcinoma; MET, metaplastic carcinoma; PAP, papillary carcinoma; HG, histological grade; HR, hormone receptor status of tumor; pos, positive; neg, negative; TN, triple negative; *ID 2007 and 2012 are sisters (ID 2007 is the index patient); ND, not determined.
