# Supplementary material for: Comprehensive Analysis of BRCA1, BRCA2 and TP53 Germline Mutation and Tumor Characterization: A Portrait of Early-Onset Breast Cancer in Brazil
Source: PLoS One. 2013 Mar 1;8(3):e57581. doi: 10.1371/journal.pone.0057581 (PMC3586086; doi:10.1371/journal.pone.0057581)
Supplement: Table S2 — Summary of the array-CGH results of fifteen tumor samples. (DOC) [file pone.0057581.s004.doc]

**Table S2.** Summary of the array-CGH results of fifteen tumor samples.

| **Patients** | **Mutational Status** | **Total CNA** | **One copy gain** | **Two or more copy gain** | **One copy loss** | **Two copy loss** |
| --- | --- | --- | --- | --- | --- | --- |
| ID_2003 | WT | 108 | 73 | 1 | 32 | 2 |
| ID_2007 | UV | 4 | 4 | 0 | 0 | 0 |
| ID_2014 | WT | 66 | 28 | 0 | 38 | 0 |
| ID_2021 | *BRCA1* | 15 | 8 | 0 | 7 | 0 |
| ID_2024 | WT | 17 | 12 | 0 | 5 | 0 |
| ID_2025 | *BRCA2* | 40 | 16 | 0 | 24 | 0 |
| ID_2026 | *BRCA1* | 22 | 15 | 0 | 7 | 0 |
| ID_2028 | WT | 3 | 3 | 0 | 0 | 0 |
| ID_2031 | *BRCA2* | 39 | 14 | 0 | 25 | 0 |
| ID_2032 | *BRCA2* | 55 | 24 | 0 | 30 | 1 |
| ID_2034 | *BRCA1* | 7 | 7 | 0 | 0 | 0 |
| ID_2036 | WT | 47 | 19 | 4 | 24 | 0 |
| ID_2037 | UV | 51 | 36 | 0 | 15 | 0 |
| ID_2038 | WT | 29 | 12 | 1 | 16 | 0 |
| ID_2048 | *BRCA2* | 53 | 22 | 0 | 31 | 0 |
