# Supplementary material for: Comprehensive Analysis of BRCA1, BRCA2 and TP53 Germline Mutation and Tumor Characterization: A Portrait of Early-Onset Breast Cancer in Brazil
Source: PLoS One. 2013 Mar 1;8(3):e57581. doi: 10.1371/journal.pone.0057581 (PMC3586086; doi:10.1371/journal.pone.0057581)
Supplement: Table S3 — Up-regulated genes in BRCA1/2-associated and -negative tumors in the enriched GO Biological Process categories. (DOC) [file pone.0057581.s005.doc]

**Table S3.** Up-regulated genes in *BRCA1/2*-associated and -negative tumors in the enriched GO Biological Process categories.

| **Genes up-regulated in *BRCA1/BRCA2*-associated tumors** | | | | | | |  | **Genes up-regulated in *BRCA1/BRCA2*-negative tumors** | | | | | | |
| --- | --- | --- | --- | --- | --- | --- | --- | --- | --- | --- | --- | --- | --- | --- |
| **Biological Function** | **Genes** | **p-value** |  | **Biological Function** | **Genes** | **p-value** |  | **Biological Function** | **Genes** | **p-value** |  | **Biological Function** | **Genes** | **p-value** |
| cell cycle | *E2F7, ASPM, PLK1, CDCA3, EXO1* | 1.77e-04 |  | mitotic cell cycle | *PLK1, CDCA8, CENPN, RRM2, BUB1* | 1.26e-04 |  | anterior/ posterior axis specification, embryo | *TBX3* | 1.18e-02 |  | muscle structure development | *TBX3, TCAP* | 2.13e-02 |
| cell cycle arrest | *PLK1, BUB1* | 4.14e-02 |  | negative regulation of cell cycle | *PLK1, BUB1* | 5.46e-03 |  | blood circulation | *HTR7, TCAP* | 1.65e-02 |  | regulation of cell development | *TBX3, SRCIN1* | 3.57e-02 |
| cell cycle phase | *ASPM, PLK1, CDCA8, CENPN, RRM2, BUB1, CDCA3, EXO1* | 9.93e-08 |  | nuclear division | *ASPM, PLK1, CDCA8, CENPN, BUB1, CDCA3* | 3.66e-07 |  | cardiac cell differentiation | *TBX3* | 4.48e-02 |  | tissue morphogenesis | *TBX3, TCAP* | 4.7e-03 |
| cell cycle process | *PLK1, RRM2, BUB1* | 1.20e-02 |  | protein modification by small protein conjugation or removal | *UBE2E3, UBE2T, PLK1* | 8.18e-03 |  | cell development | *TBX3, SRCIN1, TCAP* | 3.89e-02 |  |  |  |  |
| cellular process | *FMR,ASPM, PLK1, CDCA8, CENPN, BUB1, CDCA3* | 8.3e-03 |  | regulation of cell cycle process | *PLK1, BUB1* | 4.26e-02 |  | chordate embryonic development | *TBX3, TCAP* | 4.09e-02 |  |  |  |  |
| chromosome organization | *CDCA8, BUB1* | 1.79e-02 |  | regulation of organelle organization | *PLK1, BUB1* | 3.10e-02 |  | embryo development | *TBX3, TCAP* | 1.99e-02 |  |  |  |  |
| lymphocyte activation involved in immune response | *EXO1* | 3.49e-02 |  | response to DNA damage stimulus | *UBE2T, PLK1, EXO1* | 1.3e-02 |  | embryonic morphogenesis | *TBX3, TCAP* | 2.83e-02 |  |  |  |  |
| M phase | *PLK1, CDCA8, EXO1* | 9.33e-04 |  | somatic cell DNA recombination | *EXO1* | 4.16e-02 |  | heart development | *TBX3, TCAP* | 2.67e-02 |  |  |  |  |
| M phase of mitotic cell cycle | *ASPM, PLK1, CDCA8, CENPN, BUB1, CDCA3* | 4.37e-07 |  | somatic diversification of immune receptors | *EXO1* | 4.73e-02 |  | muscle cell differentiation | *TBX3, TCAP* | 1.26e-02 |  |  |  |  |
