# Supplementary material for: Comprehensive Analysis of BRCA1, BRCA2 and TP53 Germline Mutation and Tumor Characterization: A Portrait of Early-Onset Breast Cancer in Brazil
Source: PLoS One. 2013 Mar 1;8(3):e57581. doi: 10.1371/journal.pone.0057581 (PMC3586086; doi:10.1371/journal.pone.0057581)
Supplement: Table S4 — Up-regulated genes in BRCA1/2-associated tumors distributed in the enriched categories of the KEGG pathway. (DOC) [file pone.0057581.s006.doc]

**Table S4.** Up-regulated genes in *BRCA1/2*-associated tumors distributed in the enriched categories of the KEGG pathway.

| **KEGG Pathway** | **Genes up-regulated in *BRCA1/2*-associated Tumors** | **p-value** |  |
| --- | --- | --- | --- |
| Cell cycle | *PLK1, BUB1* | 4.36e-03 |  |
| Glutathione metabolism | *RRM2* | 4.11e-02 |  |
| Mismatch repair | *EXO1* | 1.91e-02 |  |
| Oocyte meiosis | *PLK1, BUB1* | 3.47e-03 |  |
| Progesterone-mediated oocyte maturation | *PLK1, BUB1* | 2.03e-03 |  |
