# Supplementary material for: Comprehensive Analysis of BRCA1, BRCA2 and TP53 Germline Mutation and Tumor Characterization: A Portrait of Early-Onset Breast Cancer in Brazil
Source: PLoS One. 2013 Mar 1;8(3):e57581. doi: 10.1371/journal.pone.0057581 (PMC3586086; doi:10.1371/journal.pone.0057581)
Supplement: Material and Methods S1 — Full details of methods. (DOC) [file pone.0057581.s007.doc]

**material and Methods S1**

Patients were selected on family history based on NCCN (www.nccn.org) criteria for Breast and Ovarian Cancer Syndrome and further classified as carriers of a family history of breast and ovarian cancer [FH(+)] or without family history [FH(-)]. Tumor and blood samples were collected during biopsy or breast surgery. Patients received no neoadjuvant treatment before tumor and blood collection, with the exception of patient ID_2019.

An additional group of tumor samples used in this study was derived from a cohort of 224 female patients diagnosed at ≥ 50 y and treated at Hospital AC Camargo. Formalin-fixed paraffin-embedded (FFPE) tissues were retrieved from the Hospital AC Camargo Biobank.

All FFPE tissues were tested for estrogen receptor (ER, Anti - Estrogen receptor α, ready-to-use, monoclonal rabbit anti-human antibody, SP1 clone, DAKOTM North America, Inc, Capinteria, California, USA), progesterone receptor (PR, FLEX Anti-Progesterone receptor, ready-to-use, monoclonal mouse anti-human antibody, PgR636 clone, DAKOTM North America, Inc, Capinteria, California, USA) and HER2 (FLEX Anti - c-erB-2 Oncoprotein; A0485, 1:1000, polyclonal rabbit anti-human antibody, DAKOTM Denmark A/S, Glostrup, Denmark) expression by immunohistochemistry. FISH analysis was performed to detect HER2 amplification in tumor samples with a HER2 score 2+ detected by immunohistochemistry reaction, following the American Society of Clinical Oncology and College of American Pathologists guidelines. The HER2 status was classified as positive when HER2 (score 3+) was detected by immunohistochemistry or HER2 DNA amplification was detected by FISH analysis.

**Tumor and peripheral blood samples**

Two samples of peripheral blood (5 ml) were collected, and red blood cells were lysed in 1X Tris-EDTA, pH 8.0, followed by centrifugation to separate leukocytes.

Tumor samples were collected during biopsy or breast surgery. Fresh frozen tumor samples were submitted to histological analysis, and manual dissection was performed by a pathologist. Only samples containing at least 70% malignant cells were included in the study.

**DNA and RNA purification**

Approximately 30 mg of frozen tissue was homogenized with Precellys 24® equipment (Carlsbad, California, USA). Afterward, the supernatant was used to purify total RNA with the RNeasy Mini kit (Qiagen, Venlo, the Netherlands) according to the manufacturer's protocol, and the pellet was used for DNA purification. Protein and membrane digestion was performed in digestion buffer with proteinase K (Invitrogen, Carlsbad, California, USA), and DNA was separated from the organic phase with phenol/chloroform/isoamyl alcohol (25/24/1).

Leukocyte pellets were used to purify DNA with the Puregene Genomic DNA Isolation kit (Gentra Systems, USA) according to the manufacturer’s instructions. The quantity and purity of RNA and DNA samples were assessed with a NanoDrop™ ND-1000 (Thermo Scientific, Wilmington, Delaware, USA). RNA integrity was controlled with the Agilent Bioanalyzer 2100 (Agilent Technologies, Palo Alto, California, USA). Only RNA samples with OD 260/280 >1.8 and RIN (RNA Integrity Number) >5 were further processed. DNA integrity was assessed by electrophoresis in an agarose gel and visualization with SYBR-Safe DNA gel stain (10,000X concentrated in DMSO – Invitrogen Life Technologies).

**Mutation screening of the BRCA1, BRCA2 and TP53 genes**

The *BRCA1* (U14680 or NM_007294.3), *BRCA2* (U43746 or NM_000059.1) and *TP53* (NM_000546) genes were sequenced in both the forward and reverse directions. The sequencing comprised 22, 26 and 10 coding exons for *BRCA1*, *BRCA2* and *TP53*, respectively, including intron–exon boundaries. CHEK2 (NM_007194.3) was screened for the c.1100delC mutation. First, the samples were screened for *BRCA1* and *BRCA2* mutations and *CHEK2* c.1100delC. All samples negative for pathogenic mutations were then submitted to *TP53* gene screening.

The PCR reactions contained 0.2 or 0.4 µM of each primer, 0.1 U Platinum Taq DNA Polymerase High-Fidelity (Invitrogen, Carlsbad, California, USA), 2 or 1.25 mM MgCl2 and 50 ng DNA, for a final reaction volume of 20 µl. PCR products were treated with exonuclease I and alkaline phosphatase (USB) and sequenced with the Big Dye Terminator v.3.1 Cycle Sequencing kit (Applied Biosystems) on an ABI Prism 3130xl (Applied Biosystems).

Chromatographic tracings were compared with the respective reference sequences with CLC Bio software. Nucleotide alterations were searched in the BIC Database (Breast Information Core; http://research.nhgri.nih.gov/bic, freeze October, 2012). Genes were considered as wild type when the nucleotide missense alterations were classified as with no clinical relevance in BIC database and/or as no or little clinical significance (values 1 and 2, respectively) in LOVD-IARC database. Genes were considered as unclassified variant (UV) when the nucleotide missense alterations were categorized as unknown clinical relevance in BIC and/or as uncertain in LOVD-IARC (value 3) database. In cases of disagreement between the two databases, the classification of LOVD-IARC was taken into consideration. Genes with any type of insertion or deletion or amino acid substitution that result in premature stop codons before amino acids 1853 and 3309 within the BRCA1 and BRCA2, respectively, were classified as mutated. The UVs were submitted to *in silico* prediction programs, such as SIFT (http://sift.jcvi.org/), PolyPhen (http://genetics.bwh.harvard.edu/pph/), Align-GVGD (http://agvgd.iarc.fr/agvgd_input.php) [1] and LOVD-IARC (http://brca.iarc.fr/LOVD/home.php?select_db=BRCA1; http://brca.iarc.fr/LOVD/home.php?select_db=BRCA2). The UV alterations were categorized according to the software recommendations. Nucleotide ambiguities leading to amino acid changes in the p53 protein were searched in the IARC database (International Agency for Research on Cancer; http://www-p53.iarc.fr/index.html). All detected alterations were confirmed in a second DNA sample in both the forward and reverse directions.

All detected alterations, irrespective of their classification, were confirmed in a second DNA sample in both the forward and reverse directions.

**Microarray RNA experiment and analysis**

The labeled cRNA from 49 samples (6 samples could not be assessed by microarray because no frozen tissue was available) was synthesized with the one-color microarray-based gene expression analysis - Quick Amp-Labeling Kit (Agilent Technologies, Santa Clara, USA) following the manufacturer's standard protocol. Briefly, 500 ng of total RNA and the Agilent RNA Spike-in for one color (Agilent Technologies, Santa Clara, USA) were reverse-transcribed into double-stranded cDNA with the MMLV reverse transcriptase enzyme and primed with the oligo-dT-T7 polymerase promoter sequence. The Cy3-labeled cRNA was then transcribed in vitro by T7 RNA Polymerase. The quantity and efficiency of the labeled amplified Cy3-cRNA were determined with a NanoDrop™ ND-1000 (Thermo Scientific, Wilmington, Delaware, USA). Labeled cRNAs were hybridized to the Agilent B4X44K G4112F whole human genome oligoarray (Agilent, Santa Clara, USA). Hybridizations were performed for 17h at 10 rpm and 65°C in a hybridization oven (Agilent). After hybridization, the slides were washed and scanned at 5-μm resolution with the Agilent Bundle Microarray Scanner System (Agilent). Scanned image files were visually inspected for artifacts, and the fluorescence intensities were extracted and pre-processed with Agilent Feature Extraction software (v9.5.3.1).

Flagged spots were those not found, with low intensity, saturated, non-uniform (considering spot and background), obtained by low PMT (photomultiplier tube), considered outliers when compared with other spots of the same population (replicates) or background of these populations, and positive and negative controls. Spots included in the analysis were those flagged in a maximum of three samples of the FH(+) group and three samples of the FH(-) group. Of 45,015 probes contained in the microarray, 23,765 transcripts were considered for analysis. Probes with replicates were averaged.

For microarray analysis, only the annotated genes were considered. Data were analyzed with a permuted t-test (MEV, TM4 software), and genes were considered differentially expressed when p ≤ 0.01 and fold-change ≥|2| (correction by adjusted Bonferroni method). Hierarchical clustering of samples was verified by Pearson correlation distance and complete linkage methods. Over-representation of pathways and biological process in the differentially expressed genes was determined with an integrative tool, FunNet software (Functional Analysis of Transcriptional Networks), using KEGG and Gene Ontology (GO) annotations. All microarray raw data have been deposited in the GEO public database (http://www.ncbi.nlm.nih.gov/geo), a MIAME compliant database, under accession number GSE37126 (http://www.ncbi.nlm.nih.gov/geo/query/acc.cgi?token=bzqzlaugqkeqsle&acc=GSE37126).

**Comparative genomic hybridization based on microarrays and data analysis**

Comparative genomic hybridization based on microarrays (array-CGH) was performed using a 4x180K whole-genome platform (Oxford Gene Technology, Oxford, UK), with an average spacing between probes of 18 kb. Briefly, samples were labeled with Cy3- or Cy5-dCTPs by random priming; purification, hybridization, and washing were performed as recommended by the manufacturer. Microarray images were processed with Feature Extraction software (v9.1), and data analysis was conducted with Genomic Workbench software (Agilent Technologies) and Nexus Copy Number 5.1 (Biodiscovery, El Segundo, Santa Clara, CA, USA).

Germline CNVs were assessed in genomic DNA samples derived from leucocytes. Analyses were performed in Genomic Workbench with the algorithm ADM-2, a minimum of 3 consecutive affected probes, a sensitivity threshold of 6.7, and a threshold log2 ratio >0.4 for duplications and <-0.5 for deletions). Microarray data were visually inspected for copy number imbalances within the BRCA1, BRCA2, and TP53 genes in resolution of a single probe.

In the tumor analysis, somatically acquired copy number alterations (SCNAs) were identified with Nexus software with the FASST2 segmentation algorithm, a minimum of 5 consecutive affected probes, and default settings (threshold log2 ratio of 0.2 or 1.14 for gain or high copy gain, respectively; −0.23 and −1.14 for loss and homozygous loss, respectively; signiﬁcance threshold set at 1.0E-7). A list of regions across the whole genome that exhibited an aberration meeting or exceeding the cut-off value of 50% was generated. The minimum common regions of recurrent aberrations were obtained by implementing the global frequency statistical approach of the STAC (Significance Testing for Aberrant Copy Number) method. Groups were compared with a p-value threshold of 0.05 and differential threshold of 50%.

In the DNA copy number analysis, we considered all common CNVs reported in the Database of Genomic Variants (DGV; http://projects.tcag.ca/variation/; freeze October, 2011) as irrelevant to the cancer phenotype.

**Reference**

1. Tavtigian SV, Deffenbaugh AM, Yin L, Judkins T, Scholl T, et al. (2006) Comprehensive statistical study of 452 BRCA1 missense substitutions with classification of eight recurrent substitutions as neutral. J Med Genet 43: 295-305.
